# The Complex Association between COPD and COVID-19

**DOI:** 10.3390/jcm12113791

**Published:** 2023-05-31

**Authors:** Nikhil T. Awatade, Peter A. B. Wark, Andrew S. L. Chan, SM Abdullah Al Mamun, Nurul Yaqeen Mohd Esa, Kazuto Matsunaga, Chin Kook Rhee, Philip M. Hansbro, Sukhwinder Singh Sohal, on behalf of the Asian Pacific Society of Respirology (APSR) COPD Assembly

**Affiliations:** 1 Immune Health Program, Hunter Medical Research Institute and University of Newcastle, Newcastle 2305, Australia; nikhil.awatade@newcastle.edu.au (N.T.A.); peter.wark@newcastle.edu.au (P.A.B.W.); philip.hansbro@uts.edu.au (P.M.H.); 2 Department of Respiratory and Sleep Medicine, John Hunter Hospital, Newcastle 2305, Australia; 3 Department of Respiratory and Sleep Medicine, Royal North Shore Hospital, St. Leonards 2065, Australia; andrew.chan@sydney.edu.au; 4 Northern Clinical School, Faculty of Medicine and Health, The University of Sydney, Sydney 2006, Australia; 5 Department of Respiratory Medicine & Sleep Medicine, Evercare Hospitals Dhaka, Dhaka 1229, Bangladesh; mamundr69@gmail.com; 6 Sunway Medical Centre Velocity, Kuala Lumpur 55100, Malaysia; n_yaqeen@yahoo.com; 7 Department of Respiratory Medicine and Infectious Disease Graduate School of Medicine, Yamaguchi University, 1-1-1 Minami-kogushi, Ube 755-8505, Japan; kazmatsu@yamaguchi-u.ac.jp; 8 Division of Pulmonary and Critical Care Medicine, Department of Internal Medicine, Seoul St. Mary’s Hospital, College of Medicine, The Catholic University of Korea, Seoul 06591, Republic of Korea; chinkook77@gmail.com; 9 Centre for Inflammation, Faculty of Science, School of Life Sciences, Centenary Institute and University of Technology Sydney, Sydney 2050, Australia; 10 Respiratory Translational Research Group, Department of Laboratory Medicine, School of Health Sciences, College of Health and Medicine, University of Tasmania, Launceston 7248, Australia

**Keywords:** COPD, COVID-19, ACE2

## Abstract

Chronic obstructive pulmonary disease (COPD) is significant cause of morbidity and mortality worldwide. There is mounting evidence suggesting that COPD patients are at increased risk of severe COVID-19 outcomes; however, it remains unclear whether they are more susceptible to acquiring SARS-CoV-2 infection. In this comprehensive review, we aim to provide an up-to-date perspective of the intricate relationship between COPD and COVID-19. We conducted a thorough review of the literature to examine the evidence regarding the susceptibility of COPD patients to COVID-19 infection and the severity of their disease outcomes. While most studies have found that pre-existing COPD is associated with worse COVID-19 outcomes, some have yielded conflicting results. We also discuss confounding factors such as cigarette smoking, inhaled corticosteroids, and socioeconomic and genetic factors that may influence this association. Furthermore, we review acute COVID-19 management, treatment, rehabilitation, and recovery in COPD patients and how public health measures impact their care. In conclusion, while the association between COPD and COVID-19 is complex and requires further investigation, this review highlights the need for careful management of COPD patients during the pandemic to minimize the risk of severe COVID-19 outcomes.

## 1. COPD a Risk Factor for COVID-19

### COPD as a Risk Factor for Infection and Poor Outcomes

Chronic obstructive pulmonary disease (COPD) patients and their respiratory cells, as well as animal models of experimental COPD, are more susceptible to respiratory viral infection and viral-induced exacerbations caused by influenza, rhinoviruses, and seasonal coronaviruses [1,2,3,4,5,6,7]. It has been reported that around 50% of COPD exacerbations are caused by respiratory viral infections [8], which suggests that SARS-CoV-2 may also cause exacerbations in COPD patients. Furthermore, the incidence of hospitalization and disease severity in patients with prior respiratory diseases, such as COPD, are much higher in patients with COVID-19 than with seasonal influenza [9]. This suggests that the healthcare burden associated with SARS-CoV-2-induced exacerbations in chronic respiratory diseases, such as COPD, will far outweigh the burden induced by other more established respiratory viruses [10,11,12].

It is likely that there will be unique factors that could influence the course of COVID-19 in Asia [13,14,15]. These effects are likely to be broad, encompassing population demographics, genetics, and socio-economic factors, as well as smoking and indoor and outdoor air-pollution [16,17,18,19,20]. An important question is whether individuals with COPD are at higher risk of acquiring SARS-CoV-2 infection and/or experiencing more severe clinical COVID-19 disease. To address these concerns, we conducted a PubMed search and found 66 manuscripts on COPD and infection with COVID-19, of which 38 were associated with epidemiology or prognosis, 21 with mechanisms, and 7 with management of the disease (Table 1). 

## 2. COPD and Susceptibility to COVID-19

A systematic review was conducted to evaluate the prevalence and mortality of chronic lung disease among COVID-19 patients. The review included 26 studies from China and three from the United States [31]. The pooled prevalences of lung comorbidities including asthma, COPD, and lung cancer were 3% (95% CI = 0–14), 2.2% (95% CI = 0.02–0.03), and 2.1% (95% CI = 0.00–0.21), respectively [31]. However, all of the studies included in the review were hospital-based, which may have resulted in a selection bias towards more severe disease. The authors did not claim that individuals with asthma or COPD were over-represented in these populations. This could indicate that there is no increased susceptibility to SARS-CoV-2 infection or it may reflect community behavioral change, as people with COPD have been advised of their susceptibility to viral infection in the past and throughout the pandemic. They may, therefore, benefit more from social isolation. Notably, the only published community-based screening study was from Iceland, and they did not assess co-morbid diseases [32]. Hence, the prevalence of COVID-19 in COPD patients will remain unknown until community-based screening studies are conducted that identify the role of chronic co-morbidities and susceptibility to COVID-19. 

There is a theoretical basis and emerging clinical evidence suggesting that COVID-19 results in worse clinical outcomes for COPD patients compared to non-COPD patients. Angiotensin converting enzyme 2 (ACE2) is the main host cell receptor for SARS-CoV-2, playing a crucial role in the virus’s entry into the cell and causing infection [33]. ACE2 levels are upregulated in the small airway epithelium and alveoli in older males and in COPD patients [34,35,36,37,38,39,40,41,42]. When combined with impaired innate and adaptive immune responses in COPD patients, which can delay respiratory virus clearance [37,40], it is biologically plausible that SARS-CoV-2 may more readily propagate in the lungs of COPD patients, resulting in more severe COVID-19. Indeed, poor outcomes post SARS-CoV-2 infection have been linked with COPD patients and current smokers [3,43,44]. 

Reference [26] conducted a retrospective evaluation of the impact of medical co-morbidities on the risk of serious adverse outcomes in COVID-19 patients in China. They found that a greater number of co-morbidities was associated with worse clinical outcomes, and among patients with various types of co-morbidities, COPD patients had the highest hazard ratio of 2.681 for the composite endpoint of admission to the intensive care unit, invasive ventilation, or death after adjusting for age and cigarette smoking status.

In another retrospective cohort study conducted in Republic of Korea, [22] examined 4610 patients infected with SARS-CoV-2 virus and found that those with COPD were more likely to require admission to the intensive care unit (7.1% vs. 3.7%) and mechanical ventilation (5.7% vs. 2.4%) than patients without COPD. Multi-variate analyses demonstrated that COPD was a significant independent risk factor for all-cause mortality after adjustment for age, sex, and the Charlson Comorbidity Index score (OR = 1.80, 95% CI = 1.11–2.93). However, COPD was not found to be a risk factor for respiratory failure. COPD patients had the most co-morbidities, with hypertension being the most common. The pre-existing severity of COPD did not appear to influence the clinical outcomes of COVID-19, including length of hospital stay, need for intensive care, respiratory failure, or all-cause mortality; however, the number of patients with severe COPD defined by ICD-10 code (International Classification of Diseases) without spirometry confirmation was relatively small.

Reference [23] conducted a retrospective study of COPD patients during the COVID-19 pandemic in Hubei, China, from December 2019 to March 2020. Among the 489 enrolled COPD patients, only two (0.41%) were diagnosed with confirmed COVID-19. Although the rates of acute exacerbations and hospitalization decreased during this period, the COPD mortality rate was significantly higher (2.86% vs. 0.65%). The authors also retroactively analyzed the characteristics of 821 patients with confirmed COVID-19, of whom 4.5% had pre-existing COPD. Patients with pre-existing COPD who died from COVID-19 had higher rates of coronary artery and cerebrovascular disease. The study concluded that pre-existing COPD was a risk factor for all-cause mortality in COVID-19 patients, and that the presence of COPD was associated with higher risk of all-cause mortality compared to COVID-19 patients without COPD.

Reference [45] conducted a systematic review and meta-analysis of 59 studies on the association between COPD and poor outcomes in COVID-19. The study found that COPD was associated with increased risks of hospitalization (OR = 4.23, 95% CI = 3.65–4.90, *p* < 0.0001), admission to the intensive care unit (OR = 1.35, 95% CI = 1.02–1.78, *p* = 0.03), and mortality (OR = 2.47, 95% CI = 2.18–2.79, *p* < 0.0001). However, the study also identified several important limitations of the studies, including their retrospective study design, relatively small sample sizes, and reliance on data obtained from single centers or regions. Furthermore, few studies examined the effects of COPD on COVID-19 outcomes as their primary endpoint, and the identification of COPD often relied on self-reporting or physician diagnosis, which may have introduced bias. Additionally, the impact of COPD could have been confounded by a range of demographic factors or co-morbidities.

A large study from the UK assessed the links between chronic respiratory diseases, including COPD, and COVID-19. They reviewed the links between >8.2 million people, including ~14,500 patients hospitalized for COVID-19, >1500 patients who progressed to the intensive care unit, and almost 6000 deaths. This study demonstrated that COPD patients were at increased risk of hospitalization and death (both HR = 1.54) [24]. Another US study of >11,000 patients hospitalized for COVID-19 also showed that COPD patients had higher mortality (HR = 1.27; 95% CI = 1.02–1.58), and COPD was the co-morbidity most highly associated with mortality, although obesity, diabetes, and hypertension were also independent predictors [25]. A large retrospective cohort study from China with >39,000 COVID-19 patients showed that after adjustment for age, sex, and other systemic co-morbidities, COPD (OR = 1.71; 95% CI = 1.44–2.03) and asthma (OR = 1.45; 95% CI = 1.05–1.98) but not bronchiectasis patients were more likely to reach to the composite endpoint of needing invasive ventilation, admission to the intensive care unit, or death within 30 days after hospitalization from COVID-19 [21]. Data from a New York cohort (13, 442 patients with COVID-19 attending the emergency department) showed that COPD was linked with higher risks of hospitalization (RR = 1.77; 95% CI = 1.67–1.87) and mortality (RR = 1.08; 95% CI = 0.88–1.33) [27]. Comparable results were documented in an Italian study of 1044 hospitalized patients, where increased risk of severe respiratory failure (RR = 1.81; 95% CI 1.03–3.2) was observed amongst COPD patients [28]. Data from >20,000 patients hospitalized with COVID-19 in The International Severe Acute Respiratory and Emerging Infection Consortium study showed that COPD was linked with high mortality risk (HR = 1.17; 95% CI = 1.09–1.27) [29].

The majority of studies have found that patients with pre-existing COPD and COVID-19 have worse clinical outcomes [30,46] or higher mortality risk [21]. However, biases inherent in retrospective cohort studies may have influenced these results, and prospective evaluation is necessary. Additionally, the long-term effects of COVID-19 among patients with pre-existing COPD are still largely unknown [43]. In those who have COVID-19 and seek medical attention due to illness, the role of co-morbidities such as COPD is clearer. A systematic review and meta-analysis was conducted to assess the associations of epidemiological co-morbidities with severity and prognosis, of which 61 studies with >10,000 COVID-19 cases were included. COPD was found to contribute to acute disease severity (RR = 4.20; 95% CI = 2.82–6.25), the need for admission to an intensive care unit (RR = 5.61, 95% CI = 2.68–11.76), the composite endpoint of worse clinical outcome (RR = 8.52; 95% CI = 4.36–16.65), the need for invasive ventilation (RR = 6.53; 95% CI = 2.70–15.84), and disease progression (RR = 7.48; 95% CI = 1.60–35.05) [47].

### 2.1. Cigarette Smoke, Vaping, and COVID-19 

Cigarette smoke (CS) is a major risk factor for developing COPD and has recently been identified as a predisposing factor for SARS-CoV-2 infection [24,35,48,49]. Recent evidence indicates that exposure to CS upregulates pulmonary ACE2 levels, the predominant host viral entry receptor for SARS-CoV-2 [50,51]. CS exposure also triggers the expansion of ACE2-expressing cells, which may explain the increased susceptibility of COPD patients to SARS-CoV-2 infection [52]. A meta-analysis found that smoking increased the risks of poor outcome (OR = 1.52; 95% CI = 1.16–2.00; *p* = 0.005; *I*² = 12%) and severe COVID-19 (OR = 1.65, 95% CI = 1.17–2.34; *p* = 0.004; *I*² = 11%). Current smokers were at higher risk of poor outcome (OR = 1.58; 95% CI = 1.10–2.27; *p* = 0.01; *I*² = 0%) than former/non-smokers [53]. 

Although the link between e-cigarette use and SARS-CoV-2 infection is not yet fully understood, emerging evidence suggests that nicotine alone induces ACE2 upregulation in human broncho-epithelial cells [54], indicating that e-cigarettes high in nicotine may increase the risk of SARS-CoV-2 infection and COVID-19 [55,56,57]. Preliminary findings have shown that e-cigarette users are five times more likely to be diagnosed with COVID-19, while dual users of e-cigarettes and traditional cigarettes are seven times more likely to be diagnosed [58]. Furthermore, e-cigarettes can promote lung inflammation and impair mucociliary clearance and oxidative stress, all of which can contribute to COVID-19 [59,60]. Studies suggest that chronic low levels of oxidative stress and inflammation, combined with the changes caused by a viral infection, may be responsible for the most severe forms of COVID-19 [61,62,63]. These changes can lead to high production of free radicals and the depletion of antioxidants induced by the virus [64]. 

### 2.2. Inhaled Corticosteroids and COPD

At the start of the pandemic, inhaled corticosteroids (ICSs) were thought to increase the risk of SARS-CoV-2 infection by impairing the immune response. However, observational studies have been conducted to examine whether ICSs have a protective or detrimental effect in COVID-19. A cohort study conducted in the US of 928 patients with asthma or COPD found that ICS users had no increased risk of COVID-19 [65]. Numerous studies have proposed the clinical efficacy of corticosteroids against severe and critical COVID-19, attributed to their anti-inflammatory and immunomodulatory properties [66]. In vitro studies have also demonstrated that inhaled glucocorticoids possess antiviral effectiveness via two mechanisms: by downregulating the expression of ACE2 and TMPRSS2 genes, which are crucial for viral cell entry, and by reducing the replication of SARS-CoV-2 in airway epithelial cells [41,67,68]. Furthermore, corticosteroids have been found to decrease the exacerbation rate in COPD and asthma often triggered by viral infections [69]. Early observations during the pandemic indicated that individuals with bronchial asthma and COPD who routinely use inhaled corticosteroids were less likely to be hospitalized for COVID-19 [70]. A larger cohort study from Republic of Korea included 7341 patients with PCR confirmed SARS-CoV-2 infection, out of which 114 were ICS users and 7227 were non-users. Among the 5910 hospitalized patients, 9% of ICS users and 4% of non-users died. However, this association was not significant when adjusted for factors such as age, sex, region, comorbidities, and hospital type [70].

Finally, the large UK OPENSAFELY retrospective cohort study examined asthma or COPD patients who were prescribed ICSs. While they found an association between increased mortality in asthma patients on high doses of ICSs and COPD patients on ICSs compared to those on LAMA/LABA, the authors concluded that unmeasured confounders could plausibly explain the increased risk of COVID-19-related death with increased disease severity [71]. Thus, the epidemiology does not demonstrate an increased risk of poorer COVID-19 outcomes with ICS use beyond that due to co-morbid disease severity. Those prescribed ICSs should continue their medications as clinically indicated, as they do not increase COVID-19 risk. There is also evidence that ICSs may be beneficial in COVID-19 [72,73].

### 2.3. Ethnic and Genetic Factors

The role of ethnicity has been identified as a potential risk factor for severe outcomes in COVID-19 [74], although this is debatable. A systematic review found that African and Asian people had higher rates of ICU admission and death. However, these findings may have been influenced by socioeconomic factors [74]. Additionally, a genetic variant located on chromosome 3 has been identified as a risk locus for respiratory failure with COVID-19. These variants are present in 50% of South Asians and 16% of Europeans, but they are extremely rare in sub-Saharan Africans [75]. However, a recent study by [76] suggested that genetic background does not play a significant role in the predisposition to severe COVID-19 among people with COPD. Furthermore, genetic polymorphisms of human leukocyte antigens (HLAs) have been identified to influence the susceptibility to various viral diseases, including SARS-CoV-2, MERS-CoV-2, influenza, dengue, and hepatitis B. Genetic variations in HLAs are also significantly associated with the susceptibility to and severity of COVID-19, thereby playing a crucial role in identifying groups at higher risk of COVID-19 disease [76,77,78].

Several studies have investigated the expression of ACE2 in various populations, yielding diverse findings. Chen et al. reported higher levels of ACE2 expression levels in Asian females [79]. Conversely, other studies found no significant differences in lung expression among different ethnic groups [80,81]. Additionally, genetic variants of ACE2 (HGNC:13557) that can affect its transcriptional activity have been described (e.g., rd2285666, c.439+4G>A) [82,83]. An early study discovered increased allele frequencies of variants (e.g., rs143695310) associated with higher ACE2 expression in East Asian populations, implying a potentially greater susceptibility to COVID-19 among individuals from that region [84]. A recent study indicated that the genetic determinants of the highest ACE2 expression levels were discovered in South Asian and East Asian populations, while the lowest ACE2 expression levels were identified in Africans [85]. Furthermore, Africans had the lowest TMPRSS2 (HGNC:11876) expression levels, whereas East Asians had the highest. Moreover, the study found substantial differences in TMPRSS2 expression levels between males and females [85]. In vitro studies have shown that SARS-CoV-2 infectivity varies by location in the respiratory tract, with the highest infectivity in the nasal epithelium and the lowest in bronchioles and epithelia [86]. Whether different ethnic groups exhibit varying levels of ACE2 expression in different parts of the respiratory tract and how this might impact SARS-CoV-2 infection remain unclear. 

China reported a lower prevalence of COPD among SARS-CoV-2 patients than the US, Germany, and France [87,88,89,90,91,92,93,94,95,96,97]. However, it remains unclear if this lower prevalence of COPD among COVID-19 patients was due to underdiagnosis of COPD in the study sample. Additionally, the findings did not support the notion that there are different levels of ACE2 expression among ethnic groups that contribute to COVID-19 risk. Therefore, more robust studies are required to better understand the relationship between ACE2 expression and the risk of SARS-CoV-2 infection in diverse populations. 

### 2.4. Pathology of COVID-19 in the Lungs of People with COPD

**Figure 1:**
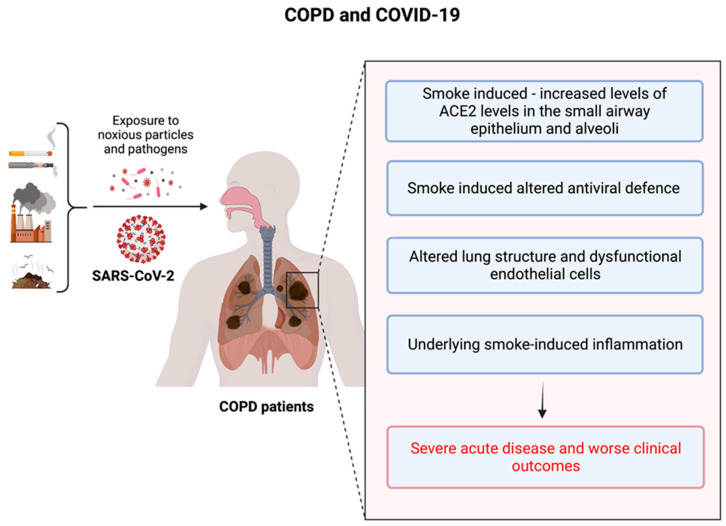
The underlying risk factors in COPD patients that are responsible for the poor outcomes during the COVID-19 crisis.

The SARS-CoV-2 ACE2 entry receptor [98,99] is upregulated in the small airway epithelium and alveoli of COPD patients [39]. Additionally, these patients exhibit impaired innate and adaptive immune responses as well as delayed clearance of respiratory viruses [37,40]. Collectively, these factors may facilitate the propagation of SARS-CoV-2 in the lungs of COPD patients, leading to rapid clinical deterioration and progression to severe COVID-19. Endothelial cell dysfunction and coagulopathy have been observed in COPD patients, with increased numbers of apoptotic endothelial cells and permeability of the airway microvasculature, which are related to airflow limitation [100,101]. Moreover, circulating levels of pro-coagulation factors are elevated in COPD patients and during exacerbations [102], which may contribute to increased risk of pulmonary emboli in these patients [103]. Consequently, COPD patients may be more susceptible to vascular damage and thrombosis during SARS-CoV-2 infection and COVID-19 [104]. We have highlighted these findings in Figure 1 below. 

## 3. Acute COVID-19 Problems in COPD Patients and Management

### 3.1. Pneumonia

SARS-CoV-2 infection-induced pneumonia differs markedly from bacterial and ventilator-associated pneumonia, which are characterized by inflammation of infected bronchioles due to polymorphonuclear leucocytes [105,106]. In COVID-19-associated pneumonia, CD8^+^ and CD4^+^ T-cells dominate in the areas around pulmonary vessels, bronchioles, and interstitial spaces [107,108]. The primary mechanism by which the virus invades host cells is the binding of SARS-CoV-2 spike (S) protein to the ACE2 receptor [109]. It infects the endothelial cells of pulmonary vessels and capillaries as well as pulmonary epithelial cells because they express high densities of ACE2 receptors. This can cause pulmonary endothelilitis with high permeability-type pulmonary edema, multiple vascular thrombosis, and neovascularization resulting from predominant intussusceptive angiogenesis [110,111]. Most COVID-19 patients experience moderate symptoms and rapid recovery, but those with COPD can develop moderate to severe COVID-19-associated pneumonia, followed by COVID-19 acute respiratory distress syndrome—ARDS (CARDS). In contrast to typical ARDS, CARDS is initially characterized by severe hypoxemia and preserved lung compliance until the development of more aggressive phases. Patients may present as clinically comfortable with “silent hypoxemia” in the early stage [112]. Dissociation between laboratory values and imaging presentation is common [113]. 

### 3.2. Oxygen and Ventilatory Support

Approximately 14% of SARS-CoV-2 patients will develop severe disease requiring oxygen therapy, and 5% will need intensive care and ventilatory support. Several ventilatory support strategies can be considered in COPD patients, depending on the type of respiratory failure (i.e., hypoxemic or hypercapnic), local practices, and resource availability. Patients with hypoxemic COPD and COVID-19 should be given controlled oxygen therapy [114]. COPD patients with severe COVID-19 should initially be maintained within a target SpO2 range of 88–92%, which can be adjusted to 94–98% following arterial blood gas (ABG) analysis that confirms the absence of hypercapnia [115]. In the absence of ABG data, all COPD patients are maintained within a target SpO2 range of 88–92%. 

Several key clinical trials have defined the important types of supportive care for people with acute COVID-19 respiratory disease, though they are not specific to people with COPD. This includes adopting awake prone positioning for patients requiring prolonged oxygen [116], which reduces the risk of needing mechanical ventilation. In patients with severe COVID-19, high-flow humidified oxygen reduced the need for mechanical ventilation compared to the conventional oxygen delivery [117]. A randomized control trial of patients requiring an FiO_2_ of 0.4 or greater also showed that treatment with continuous positive airways pressure (CPAP) and oxygen was superior to both high-flow humidified and conventional oxygen in reducing the need for mechanical ventilation [118].

While the role of non-invasive ventilation in the context of acute hypoxic respiratory failure is less clear, observational studies have shown it to be effective [119,120]. In contrast, its role in patients with acute hypercapnic respiratory failure and COPD is well established. Although its effectiveness in severe COVID-19 has not been demonstrated, non-invasive ventilation is still recommended for individuals with hypercapnic respiratory failure and COPD who also have COVID-19 [121]. Initially, there were concerns that high-flow humidified oxygen and non-invasive ventilation might spread potentially infectious aerosols; however, there is no evidence to suggest that these interventions are more likely to cause infection than coughing [122].

## 4. Post COVID-19 Syndrome

### 4.1. Post COVID-19 Risks for COPD Patients 

The progression of the COVID-19 pandemic has revealed that some patients continue to experience multi-systemic clinical features and complications beyond the initial period of acute infection and illness. COPD patients have been identified as a high-risk group for severe post-COVID-19 syndrome [43]. Persistent symptoms and/or delayed or long-term complications of SARS-CoV-2 infection beyond four weeks are currently referred to as post-acute or long COVID-19 [123]. Recent reviews have further categorized it into two groups: (1) symptoms and clinical features present from 4–12 weeks beyond acute COVID-19 are termed subacute or ongoing symptomatic COVID-19; (2) symptoms and clinical features that persist or present beyond 12 weeks of the onset of acute COVID-19 and are not attributable to alternative diagnoses are termed long COVID-19 [124,125]. 

### 4.2. Post COVID-19 and COPD Exacerbations

The definition of an exacerbation is the worsening of COPD symptoms leading to the need for additional pharmacological treatment [95]. Among the poor outcomes for COPD patients with frequent exacerbations are reduced lung function and high mortality rate [126,127]. The common causes of COPD exacerbations include viral [128,129,130,131] and bacterial [40,129] infections [7]. During the current COVID-19 pandemic, SARS-CoV-2 virus infection is one of the likely causes of acute COPD exacerbations. While seasonal causes of acute COPD exacerbations include coronaviruses, it remains controversial whether COVID-19 and long COVID-19 in a COPD patient should be considered an exacerbation or not. Based on our current definition of an exacerbation as a clinical diagnosis based on worsening symptoms requiring a change in treatment [132], a COPD patient with COVID-19 and/or long COVID-19 presenting with worsening cough and dyspnea would meet the requirements. However, the pathophysiology of a typical COPD exacerbation is very different from COVID-19-associated pneumonia and long COVID19, based on imaging and post-mortem features [110,133]. COVID-19 and long COVID-19 in a COPD patient likely involve different pathological processes.

### 4.3. What Are the Treatments for COPD Patients with COVID-19 and Long COVID-19?

Considering underlying COPD is crucial when treating COVID-19 and long COVID-19 in COPD patients, as their features differ from typical of COPD exacerbations. Even after diagnosing COVID-19 and long COVID-19 in COPD patients, a concomitant exacerbation requiring treatment cannot be ruled out. Antibiotics and bronchodilators are often prescribed for COPD exacerbations, but whether and how these therapies should be administered to COPD patients during the pandemic remain unanswered questions.

Not all COPD exacerbations need to be treated with antibiotics [132], which should be reserved for exacerbations that require hospitalization or ventilatory support based on current guidelines [134]. Occasional concomitant bacterial infections have been reported in COVID-19, as shown in a recent meta-analysis where 8% of COVID-19 patients had bacterial or fungal co-infection [135]. Increasing severity of COVID-19 is associated with increased risk of co-infection, as demonstrated in a cohort study that reported that 50% of COVID-19 non-survivors experienced secondary infections and 31% had ventilator-associated pneumonia [60]. Considering the difficulty in distinguishing SARS-CoV-2 infections from bacterial pneumonia and the high risk of bacterial infections in COPD patients, local/national pneumonia guidelines recommend treating hospitalized COPD patients with COVID-19 and long COVID-19 with broad-spectrum antibiotics. This follows WHO treatment guidelines for severe COVID-19 and long COVID-19 [136]. Performing microbiological analysis, such as sputum culture, upon hospital admission for exacerbated COPD patients and stopping antibiotics in the absence of co-infection is reasonable [137]. However, clinical data on bacterial co-infections in COVID-19 and COPD patients are lacking, and more research is needed to determine the role of antibiotics in treating COVID-19 patients with COPD exacerbations.

Hospitalized COPD patients experiencing exacerbations are often prescribed nebulized bronchodilators, although pressurized metered-dose inhalers (pMDI) used with a spacer are an alternative mode of inhalation. pMDIs have been shown to be non-inferior to nebulizers in exacerbation management [138]. Some long-acting dual bronchodilators have rapid onset of action, are more effective, and last longer, making them a preferred option [139]. High doses of nebulized short-acting bronchodilators are often administered for COPD exacerbations, and there is no maximum specified dose. It is advisable to double the maximum maintenance dose of long-acting bronchodilators to make it equivalent to the high doses of short-acting bronchodilators for COPD exacerbations. Bronchodilators administered via pMDI and spacer are recommended over nebulizer treatment in symptomatic exacerbated COPD patients with COVID-19 and long COVID-19, since the safety of nebulizers is still controversial. 

### 4.4. Rehabilitation & Recovery of COPD Patients with Long COVID-19

Considerable morbidity is experienced by patients with severe COVID-19 during hospitalization, including lethargy, dyspnea, diffuse myalgias, and cognitive dysfunction, which may persist even after recovery from acute illness [140,141]. After hospital discharge, 50% of patients with severe COVID-19 experience worsened dyspnea, impaired exercise tolerance, and may benefit from pulmonary rehabilitation (PR) [142,143,144]. Patients with underlying COPD may experience more pronounced post-COVID-19 complications due to underlying structural lung damage. Multiple randomized controlled trials, meta-analyses, and evidence-based reviews provide strong evidence of the benefits of PR in symptomatic COPD patients [145]. PR is one of the most effective treatment strategies for improving COPD patients’ dyspnea, health status, and exercise tolerance. It may also help to reduce anxiety and depressive symptoms. However, due to social restrictions, physical separation, and concerns about SARS-CoV-2 community transmission, conventional PR cannot be easily performed during the pandemic.

The pandemic has had various impacts on COPD patients. Physical clinic and home visits have been reduced along with PR sessions. As a result, many COPD patients have stayed at home despite experiencing severe exacerbations, leading to delayed treatment and poor outcomes, which is similar to what has been observed for other diseases such as myocardial infarction [146,147]. Adapting to new healthcare norms involves expanding telehealth and virtual clinics. Multiple randomized controlled trials have shown that telehealth for COPD patients is non-inferior to usual care in terms of exacerbations, hospital admissions, and quality of life [148,149,150,151,152]. Online PR program sessions appear to be just as effective as in-person sessions [153,154,155]. Establishing virtual programs is encouraged to ensure that COPD patients receive optimal care despite social distancing measures [156]. In-person PR should not occur when the community prevalence of COVID-19 is high because COPD patients are vulnerable to severe complications of COVID-19 [151,152]. However, in-person PR may be considered when the community spread of COVID-19 is low. It is important to note that social exercise, especially indoors, is a high-risk activity for COVID-19 transmission [157]. 

### 4.5. Vaccination and COPD

Several vaccines against COVID-19 have been developed and have shown to be effective. A subgroup analysis of a recent phase 3 randomized trial showed that the mRNA-1273 vaccine was equally effective in preventing COVID-19 in subjects with and without risk factors for severe disease, including chronic lung disease [158]. However, due to their rapid development, evidence regarding the efficacy and potential adverse effects of these vaccines for different demographic is still lacking. Therefore, further studies are needed to determine their effectiveness and safety among people with COPD and other comorbidities. Studies have investigated the correlations between influenza vaccination, susceptibility to SARS-CoV-2 infection, and outcomes of COVID-19. Influenza vaccination has been found to be independently associated with a lower risk of mortality at 60 days in COVID-19 patients (OR = 0.2; 95% CI = 0.082–0.510) [159]. Another study in the UK showed reduced odds of all-cause mortality (OR = 0.76; 95% CI = 0.64–0.90) in COVID-19 patients [160]. The underlying molecular mechanisms are unknown, but we can speculate that the flu increases ACE2 receptor expression in pulmonary alveolar cells, which may worsen a subsequent SARS-CoV-2 infection [161]. Preventing influenza with a vaccine may lower the viral load and severity of COVID-19. However, an Italian study found no association between death or hospitalization and the flu vaccine [162]. Given this evidence, it is recommended that COPD patients receive vaccination against both seasonal flu and/or COVID-19 to minimize their risks of severe disease and mortality.

### 4.6. Influence of Public Health Measures on Care for COPD Patients in the Pandemic

Public health measures, such as social distancing, lockdown, and reduced face-to-face consultations during the pandemic, have had a significant impact on healthcare-seeking behavior and access to healthcare. Hospitalizations for COPD exacerbations decreased during the pandemic due to reduced infection exposure during social isolation [163,164]. However, the impact of these public health measures on the quality of care and control of chronic conditions requires careful evaluation. 

The recent surveys conducted in China and Spain provide valuable insights into the impact of the COVID-19 pandemic on COPD patients. The survey of 153 COPD patients in China showed that most continued to use their inhaled medications as before the pandemic, with only 30% experiencing worsening respiratory symptoms [165]. Of those who experienced symptoms, 55.5% did not seek medical attention due to fear of contracting the virus, while 28.8% managed mild symptoms on their own. In Spain, a study of 100 patients conducted by telephone interview found that 90% of patients had medical consultations or complementary tests cancelled during lockdowns [166]. However, approximately 60% had a medical visit by telephone and reported a high degree of satisfaction. Notably, 63% of patients considered their lung health status to be the same as before lockdown, and 19% even felt better. Taken together, these surveys suggest that public health interventions during the pandemic can help to control COPD and that telemedicine may be an effective way to provide medical care for COPD patients when in-person visits are not possible.

The Global Initiative for Chronic Obstructive Lung Disease (GOLD) recently introduced a tool to support remote follow-up for COPD patients [167]. This tool encourages standardized evaluation and documentation, ensuring high-quality patient follow-up. Clinicians and policymakers are interested in the potential impact of applying this tool or similar ones to patient care. Therefore, we encourage further reports to inform future recommendations and health policies.

## 5. Conclusions

The balance of evidence strongly suggests that COPD patients are more susceptible to contracting COVID-19, developing severe acute disease, and have worse clinical outcomes. However, the heterogeneity of the COPD population across different countries with varying proportions of co-morbidities may complicate assessing the net effect of COPD on COVID-19-related outcomes due to variability in testing and admission strategies. Additionally, individuals with COPD who have pre-existing exercise intolerance and cardiovascular morbidities are more likely to experience a complicated recovery, placing them at greater risk during the COVID-19 pandemic.

## Figures and Tables

**Table 1:** Summary of COPD studies. ND: Not defined.

Authors	Country	Number of Patients	Study Type	Study Setting	Odds Ratio (OR)	Confidence Interval (CI)	Reported Outcomes
Guan et al., 2021 [21]	China	39,420 COPD (56.6%)	Retrospective case series	Hospital (multi-center)	1.71	1.44–2.03	CRD was associated with poor clinical outcomes of COVID-19
Lee et al., 2021 [22]	Republic of Korea	4610 COPD: 141 (3.1%)	Retrospective cohort	Hospital (multi-center)	1.80	1.11–2.93	COPD was not a risk factor for respiratory failure
Hu et al., 2020 [23]	China	489 COPD	Retrospective cohort	Hospital (single-center)	ND	ND	COVID-19 patients with pre-existing COPD had high risk of all-cause mortality
Aveyard et al., 2021 [24]	UK	8,256,161 COPD: 89,605 (46.3%)	Population cohort	Hospital (multi-center)	1.54	1.45–1.63	COPD patients were at increased risk of hospitalization and death
Girardin et al., 2021 [25]	US	11,512	Case series	Hospital (nationwide)	1.27	1.02–1.58	Higher mortality risks were associated with a history of COPD
Guan et al., 2020 [26]	China	1590	Retrospective case study	Hospital (nationwide)	2.681	1.42–5.04	Highest hazard ratio in COPD patients
Kalyanaraman Marcello et al., 2020 [27]	US	13,442	Retrospective cohort	Hospital (nationwide)	1.77	1.67–1.87	COPD was linked with higher risk of hospitalization and mortality
Bartoletti et al., 2020 [28]	Italy	1044	Retrospective cohort	Hospital (multi-center)	1.81	1.03–3.2	Increased risk of severe respiratory failure was observed amongst COPD patients
Docherty et al., 2020 [29]	UK	20,133	Observational cohort study	Hospital (multi-center)	1.17	1.09–1.27	COPD was linked to high mortality risk
Hansen et al., 2021 [30]	Denmark	5104 COPD: 432	Retrospective cohort	Hospital (nationwide)	21.2	18.8–23.6	Patients with COPD had slightly increased risk of developing severe outcomes of COVID-19

## Data Availability

Not applicable.
